# Why We Must Stop Assuming and Estimating Menstrual Cycle Phases in Laboratory and Field-Based Sport Related Research

**DOI:** 10.1007/s40279-025-02189-3

**Published:** 2025-03-14

**Authors:** Kirsty Jayne Elliott-Sale, Marco Altini, Patricia Doyle-Baker, Eva Ferrer, Tessa Rose Flood, Rachel Harris, Franco Milko Impellizzeri, Xanne Janse de Jonge, Katrine Okholm Kryger, Gary Lewin, Constance M. Lebrun, Alan McCall, Sophia Nimphius, Stuart M. Phillips, Paul A. Swinton, Madison Taylor, Evert Verhagen, Richard James Burden

**Affiliations:** 1 Department of Sport and Exercise Sciences, Institute of Sport, Manchester Metropolitan University Institute of Sport, Manchester, M1 7EL UK; 2 Department of Human Movement Sciences, Vrije Universiteit Amsterdam, Amsterdam, The Netherlands; 3 Human Performance Lab, Faculty of Kinesiology, University of Calgary, Calgary, Canada; 4 Barça Innovation Hub, Health & Wellness Area, Barcelona, Spain; 5 Sports and Exercise Medicine Unit, Hospital Clinic and Sant Joan de Déu, Barcelona, Spain; 6 Australian Institute of Sport, Bruce, ACT Australia; 7 Perth Orthopaedics and Sports Medicine Research Institute, Perth, WA Australia; 8 School of Sport, Exercise and Rehabilitation, Faculty of Health, University of Technology Sydney, Sydney, NSW Australia; 9 Griffith Sports Science, School of Health Sciences and Social Work, Griffith University, Gold Coast, QLD Australia; 10 Medical and Anti-Doping Unit, Football Division, UEFA, Nyon, Switzerland; 11 Arsenal Women’s Football Club, London, UK; 12 Department of Family Medicine, University of Alberta, Edmonton, AB Canada; 13 Arsenal Performance and Research Team, Arsenal Football Club, London, UK; 14 School of Medical and Health Sciences, Edith Cowan University, Joondalup, Australia; 15 Department of Kinesiology, McMaster University, Hamilton, ON Canada; 16 School of Health Sciences, Robert Gordon University, Aberdeen, UK; 17 School of Sport Sciences, UiT The Arctic University of Norway, Tromsø, Norway; 18 IOC Research Centre for Prevention of Injury and Protection of Athlete Health, Department of Public and Occupational Health, Amsterdam Collaboration on Health and Safety in Sports, Amsterdam Movement Sciences, Amsterdam UMC, Amsterdam, The Netherlands; 19 United Kingdom Sports Institute, Manchester, UK

## Abstract

The increased growth, popularity, and media interest in women’s sport has led to calls for greater prioritisation of female-specific research and innovation. In response, science and medicine researchers have increased the volume of sport-related studies investigating female-specific matters, such as the menstrual cycle. Whilst the accelerated rate of published studies with female participants is welcome, the emerging trend of using assumed or estimated menstrual cycle phases to characterise ovarian hormone profiles is a significant concern. Replacing direct measurements of key characteristics of the menstrual cycle (e.g. the surge in luteinising hormone prior to ovulation via urine detection and sufficient luteal phase progesterone via blood or saliva sampling) with assumptions or estimates (i.e. no measurements) is proposed to be a pragmatic and convenient way of generating data, particularly in field-based research (i.e. elite athlete environments), where time, resources, and athlete availability are sometimes constrained. Using assumed or estimated phases, however, amounts to guessing the occurrence and timing of ovarian hormone fluctuations and risks potentially significant implications for female athlete health, training, performance, injury, etc., as well as resource deployment. The positive intentions of researchers and scientific journals in this space are not in question. The aim of this Current Opinion is to explain why using assumed or estimated menstrual cycle phases is an approach that has little scientific basis and lacks the rigour and appropriate methodological quality to produce valid and reliable data. In doing so, we provide evidence-based responses to common speculation points and offer recommendations for future research.

## Key Points

**Table:** 

Assumptions and estimations are not direct measurements and, as such, represent guesses, which should be avoided in laboratory and field-based sport-related research.
Assuming or estimating menstrual cycle phases is neither a valid (i.e. how accurately a method measures what it is intended to measure) nor reliable (i.e. a concept describing how reproducible or replicable a method is) methodological approach.
Extra caution should be exercised when drawing conclusions from data linked to assumed or estimated menstrual cycle phases.
Transparent and honest reporting of the limitations associated with assumptions and estimations, as well as the implications of these limitations, must be provided and justified.

## Introduction

Numerous recent studies (e.g. [1–14]) have assumed or estimated menstrual cycle phases while measuring various aspects of training, performance, and/or injury surveillance. We commend their efforts to provide insight into a complex area of exercise physiology (i.e. reproductive and non-reproductive functions of menstrual cycle phases), particularly in elite athlete groups, wherein research is challenging. We question, however, the science behind an assumed or estimated cycle phase approach, namely the validity and reliability of assuming or estimating menstrual cycle phases; inferences drawn from data linked to assumed or estimated menstrual cycle phases; and the repercussions of this low-quality evidence on applied practice. We recently published an editorial [15] briefly describing issues associated with assumed and estimated menstrual cycle phases, calling for reviewers and editors to insist upon direct measurements rather than guesses (i.e. assumptions and estimations) and honest, transparent reporting when assumed or estimated phases are employed. Herein, we provide a fuller critique of an assumed or estimated menstrual cycle phase approach in research studies; the interpretive value such an approach provides; and the inherent risks this approach presents to evidence-informed practice. We also describe how researchers can avoid these issues in the future and undertake higher-quality approaches to their sport-related laboratory and field-based studies examining menstrual cycle phases. Although we provide examples of studies employing an assumed or estimated approach to menstrual cycle phases, the intention of this Current Opinion is not to degrade studies, but rather to encourage researchers to reflect upon current standards and practices associated with menstrual cycle phase determination. Hence, the aim of this Current Opinion is to place ‘menstrual cycle tracking for research’ in perspective, given that a consensus has not yet been reached despite current international interest. This paper does not cover ‘menstrual cycle tracking for health or performance monitoring’ in applied settings (i.e. elite sport). This topic warrants a comprehensive review of current evidence and practices, alongside a thorough needs assessment, and as such is outside the scope of this current paper. As research is often intended to bridge science to practice, we have engaged a diverse, interdisciplinary group of academic researchers plus research-active practitioners and clinicians to provide insight from both laboratory and field-based studies.

## Physiology

The menstrual cycle is characterised by three inter-related cycles; ovarian, hormonal, and endometrial. In brief, the ovarian cycle refers to the lifecycle of an oocyte; the hormonal cycle represents the fluctuations in ovarian hormones; and the endometrial cycle describes the changes in the lining of the uterus. The guidance provided herein relates to measurements associated with the hormonal (e.g. concentrations of ovarian and pituitary hormones via blood, urine, or saliva samples) and endometrial (e.g. bleeding patterns) cycles only as these are most commonly used in sport-related research, with a clear emphasis on the importance of measurements rather than assumptions or estimations. Measurements related to the ovarian cycle are not typically used outside of clinical settings; for example, the only way to definitively know if ovulation has occurred is with direct ultrasound; however, transvaginal ultrasonic visualisation is infinitely challenging even in controlled clinical settings. We acknowledge that a parameter of interest can be measured in a variety of ways and that all measurements are open to debate, but nevertheless these are still measurements and nothing is guessed. Particular attention should be paid to the accuracy, sensitivity, and variability of the hormonal analyses used in cited studies as these data provide critical context for evaluating the reliability of the research findings.

**Fig. 1:**
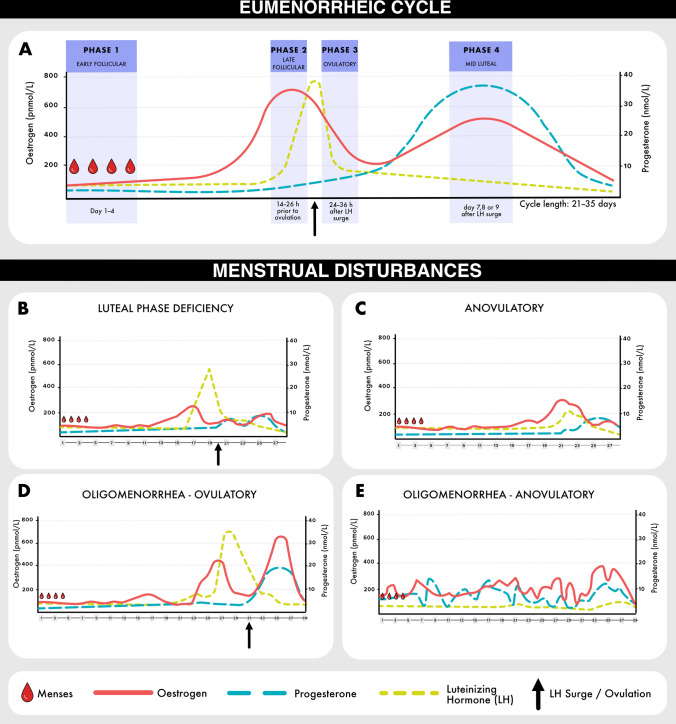
The ovarian hormone profile of a eumenorrheic cycle (**A**), luteal phase deficient cycle (**B**), anovulatory cycle (**C**), and ovulatory and anovulatory oligomenorrheic cycles (**D** and **E**). Panels **B–E** are adapted from Allaway et al. [17] with permission

Herein, for the purpose of research studies, we have adopted the definition of a eumenorrheic cycle (i.e. a healthy menstrual cycle; Fig. 1) as previously described by Elliott-Sale et al. [16]; namely a eumenorrheic menstrual cycle is characterised by cycle lengths ≥ 21 days and ≤ 35 days, resulting in nine or more consecutive periods per year, evidence of a luteinising hormone surge, and the *correct* hormonal profile. Whilst guidance on the *correct* hormonal profile is provided in Elliott-Sale et al. [16], it should be noted that there are inconsistencies in the literature in phase determination based solely on hormone levels and, as such, all studies should decide a priori upon their hormonal phase-based boundaries and clearly define these within their methodology. As we are interested in the hormonal effects of the menstrual cycle on a parameter of interest, we describe the cycle as four hormonally discrete phases, based on changes in endogenous oestradiol and progesterone levels, while noting that the cycle could be divided based on other factors (e.g. a phase to represent pre-menstrual syndrome based on symptomology). Based on these criteria, the presence of menses and an average cycle length of 21–35 days does not guarantee a eumenorrheic hormonal profile. Simply, this means the calendar-based method of counting days between one period and the next cannot be relied upon to determine a eumenorrheic menstrual cycle and should not be used to classify subsequent (i.e. phases 2, 3, and 4) cycle phases in research studies. Allaway et al. [17] eloquently illustrated (Fig. 1) how, when cycles are assessed solely based on regular menstruation and/or cycle length, subtle menstrual disturbance, such as anovulatory or luteal phase deficient cycles, can go undetected, despite presenting with meaningfully different hormonal profiles. Note that subtle menstrual disturbances are often asymptomatic but are potential precursors to more severe disturbances, such as amenorrhea. Given the high prevalence (up to 66%) of both subtle and severe menstrual disturbances reported in samples of exercising females [18], these disturbances should be evaluated. For reference, women who regularly menstruate, with menstrual cycle lengths ≥ 21 days and ≤ 35 days, but without confirmed ovulation or sufficient progesterone should be referred to as ‘naturally menstruating’ in research studies [16].

**Fig. 2:**
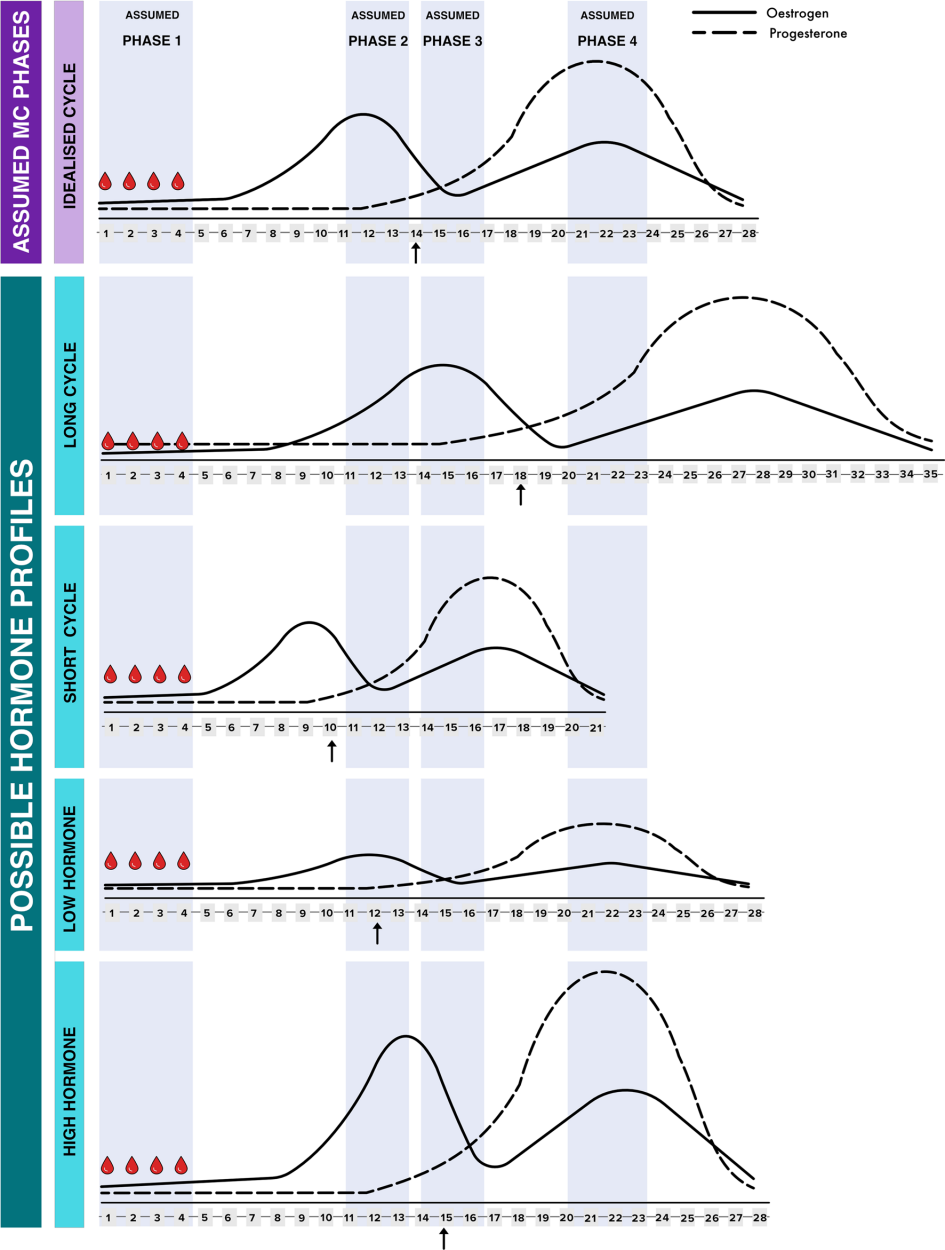
Conceptual illustration highlighting the misalignment between assumed versus actual ovarian hormone profiles. The *upwards arrow* denotes anticipated ovulation. *MC* menstrual cycle

It was recently stated that ‘Because menstruation (onset of menstrual bleeding) is a clear-cut point, this phase is easily determined, as is the premenstrual phase, which is just before the onset of menstruation’ [9]. Whilst the pre-menstrual phase is indeed ‘just prior to the onset of menstruation’, it cannot be assumed the hormonal profile during the pre-menstrual phase is universal. The occurrence and timing of ovulation and sufficient progesterone determine the ovarian hormone profile in phase 4. As such, the pre-menstrual phase still represents an assumed rather than a ‘clear-cut’ hormonal phase (Fig. 2).

Terminology and context are critical when using the terms ‘eumenorrhea’ or ‘naturally menstruating’ to describe participant cycles in research. The term ‘naturally menstruating’ should be applied when a cycle length between 21 and 35 days is established through calendar-based counting, but no advanced testing is used to establish the hormonal profile. We acknowledge that the calendar-based approach can be useful when no other resources are available to establish a ‘naturally menstruating’ profile, wherein women experience menstruation, with cycle lengths between 21 and 35 days. In this situation, the cycle can only be split into menstruation and non-menstruation days. Phase names or numbers cannot be reliably attributed to non-menstruation days without advanced testing. The calendar-based counting approach excludes severe menstrual disturbances (e.g. amenorrhea) but cannot detect subtle disturbances, thereby providing limited information on hormonal status. Thus, it can only be used to compare an outcome during menstruation (i.e. typically 3–7 days) against the remaining days of the cycle (typically 14–28 days). This is problematic because it only provides dichotomised continuous data. The term ‘eumenorrhea’ and/or specific phase names should be reserved for situations in which menstrual function has been confirmed through advanced testing.

## Terminology and Context

In research, assumptions are ‘beliefs’ (i.e. axioms and postulates) taken for granted and that constitute the premises under which testable implications can be examined [19]. Even if some assumptions are not formally tested, they should be reasonable, plausible, and logically consistent; otherwise the resulting conclusions will be invalid [19, 20]. In research, an estimate is an ‘informed best guess’ (i.e. reasonable attribution) of the true (population) value; the magnitude of the discrepancy between the true value and the estimate should be as small as possible (i.e. accurate) to make the findings meaningful and useful [21, 22]. This estimation can be based on direct measures of the variable of interest (i.e. direct estimation) or indirect information (i.e. indirect estimation). An indirect estimation is inevitably based on more assumptions than direct estimations and the validity of these assumptions defines the conditions under which this estimation is valid. If these additional assumptions are not reasonable and are violated, the estimation is not valid [23]. Herein, we are specifically concerned about the indirect estimations used in some menstrual cycle phase research (e.g. [1–14]) and why the assumptions they are based on are not reasonable. We acknowledge the ongoing consideration given to the most appropriate direct measures/estimations for menstrual cycle phase determination. As such, ‘estimations’ is used throughout to refer to indirect estimations and ‘direct measures’ is used throughout to refer to direct estimations.

Researchers should understand that when cycle phases are assumed and estimated solely based on regular menstruation, several guesses are implicitly made; i.e. ovulation and sufficient phase 4 progesterone are believed to be present and occurring at a set time. As such, assumed and estimated phases involve two distinct factors that compound each other, namely occurrence (i.e. that they happened) and timing (i.e. when they occurred). Moreover, the approach of separating the cycle into even more assumed phases based on assumed hormonal profiles to improve sensitivity in identifying meaningful changes is inadvisable. These additional assumptions and estimations are likely to further increase the chance of misrepresented and misinterpreted datasets because these assumptions are often not reasonable and plausible.

An indirect estimation represents a guesstimate (i.e. ‘an attempt to calculate something that is based more on guessing than on information’ [24]) approach to research, wherein the validity of the estimation depends on the validity of the assumptions on which it is based. We know, however, that these assumptions are—more often than not—incorrect, potentially leading to flawed conclusions and decisions. Therefore, we recommend that researchers follow the guidance outlined by Janse de Jonge et al. [25] and Elliott-Sale et al. [16] for verifying (‘making sure or demonstrating that [something] is true, accurate, or justified’ [26]) cycle phases in research studies.

*Tracking* is ‘the act or process of following something or someone.’ An assumed phase approach tracks one phase of the menstrual cycle only (i.e. menses, also known as phase 1 or menstruation). In research studies, cycle tracking intending to define cycle phases should incorporate all of the characteristics defined by eumenorrhea, thereby providing the key phases associated with major fluctuations in endogenous oestrogen and progesterone, such as phases 2 and 4, otherwise known as pre-ovulatory and mid-luteal phases [16].

## Transparency of Reporting

Whilst some limitations associated with assumed or estimated cycle phases are described in such studies, their true impact on interpretation, degree of certainty, and application of their findings is never reported. Transparency of reporting in this area must be improved so readers can genuinely understand the pitfalls associated with a lack of menstrual cycle phase verification; several examples are provided in Table 1. Moreover, studies should justify assumptions and estimations, especially if repeatedly used, and clearly acknowledge the limitations associated with their methods.

**Table 1:** Summary table describing pitfalls associated with using assumed or estimated menstrual cycle phases and how to avoid them

Refrain from	Example	Implication(s) associated with assumption/estimation	Do instead
Using the incorrect definition to describe participants	Using eumenorrheic when it should be naturally menstruating (when ovulation and sufficient luteal phase progesterone have not been confirmed)	The sample might include participants with subtle menstrual disturbances, meaning they are not eumenorrheic, and as such have been misclassified	Use correct terminology to reflect the endocrine/physiological profile of the participants
Using discrete phase names when these phases have not been verified	Using phase number (e.g. phase 4) or names (e.g. mid-luteal) for the non-menstruation days when an assumed approach has been taken	The sample might include participants with subtle menstrual disturbances, meaning the assumed phases have been misclassified. Participants may not be in the assumed phase due to variability within the cycle	Establish phases using advanced biological direct measurements or if this is not possible, use the terms menstruation and non-menstruation days
Inaccurate reporting of phase names or numbers	If a study has measured the luteinising hormone surge but not progesterone levels, but still uses the terms phase 4 or mid-luteal phase	The sample might include participants with luteal phase deficiency, meaning the assumed phase has been misclassified. Participants may not be in the assumed phase due to variability within the cycle	Use ‘assumed luteal phase’
Associating certainty with assumed or estimated phases (i.e. inferring causality)	X outcome was significantly associated with X (assumed) phase of the menstrual cycle	The outcome might be associated with a hormonal profile that does not exist, which is misleading to the end-user	State that assumptions and estimations are guesses and not direct measurements, and list all the implications of these guesses
Ambiguity with the term tracking	Stating the menstrual cycle was tracked, when only menstruation was tracked	The remaining phases, outside of menstruation/phase 1, cannot be tracked using an assumed or estimated approach, meaning that the term tracking has been overstated	Set the scope of your tracking: describe if menstruation was tracked or if menstrual cycle phases were tracked, as these are not synonymous
Repeatedly listing the same avoidable limitations	Using assumed phases in numerous studies	Continually contributing low-quality data regardless of study setting (e.g. the possibility of a high-quality approach) or research/applied standards	Evaluate the possibility of improving methodological quality, employ a high-quality approach when possible, and engage with published guidelines in this area
Listing limitations without full disclosure of their impact	Stating that best practice for menstrual cycle phase determination has not been used, without stating the impact of assumed or estimated phases	Assuming that readers know the full extent of implications of assumed or estimated phases on the degree of certainty in the findings and their potential application	Be transparent in reporting the limitation, leaving no ambiguity around methodological quality and subsequent impact of their work
Using flawed or low-quality studies to justify an assumed or estimated approach	Referencing ‘one-off’ studies that are not fit-for-purpose to justify an assumed or estimated approach	By providing a reference to support an approach the end-user might be misled into thinking the approach taken is credible, as it has been published in a peer-reviewed journal	Be honest about the quality of the study being used to justify the approach taken; list its limitations and implications of those limitations
Using pragmatism or unfounded statements to justify methodological shortcomings	Stating that elite female athletes will not provide urinary, blood, or salivary samples to verify menstrual cycle phases	These statements underserve female athletes by implying that direct measurements, rather than assumptions and estimations, are not possible, which is incorrect (i.e. one study/sample cannot speak for all studies/samples)	Accurately represent the confines of the current study; state if certain resources were unavailable or if participants were unwilling to do X or Y, without inferring this is the case for all elite sport or all studies with female athletes
Using the term preliminary to justify methodological shortcomings	Stating that findings are preliminary without describing how and why they are preliminary, and what future plans are to expand the work	The term preliminary means preceding or done in preparation for something fuller or more important, which is misleading if it is a stand-alone study (i.e. it validates a lesser approach)	Clearly state how and why the work is preliminary and how it will be expanded, or do not use this term if it does not accurately reflect the situation
Overstating findings	Using text snippets as soundbites, which could be misleading, rather than being fully accurate and transparent with all reporting	Overstated findings have many implications including unsupported changes to practice, misleading athletes and their entourage, as well as unnecessary changes to policies or funding, causing unwarranted anxiety to athletes and their entourage, etc	Take responsibility for the integrity of reporting of findings both within the publication and in other media—whilst we acknowledge that others might misquote an interview or publication, we should use the channels available to us to correct such instances

We commend all researchers who publish individual datasets related to verified menstrual cycle phases, as direct measures have been made and not approximated and are therefore fit-for-purpose. This practice is in line with the Open Science Framework as these data highlight the within- and between-variability (Table 2) associated with menstrual cycle characteristics, which are inevitably overlooked by an assumed or estimated approach. Individual datasets clearly show that the population criteria (e.g. eumenorrhea) have been met, while assumed or estimated datasets cannot. Dam et al. [27], Colenso-Semple et al. [28], and Taylor et al. [29] have shown compelling data illustrating discrepancies between ‘assumed’ versus ‘verified’ phases (i.e. the menstrual cycle reality of the participants). As such, we encourage all study investigators to adopt this research practice (i.e. verifying phases and including individual datasets), as the assumed and estimated approach is a threat to the study’s validity that should be avoided. At the very least, these must be transparently addressed and acknowledged within publications (including the abstracts) as a limitation, ensuring that the conclusions are not overstated and do not contain unsupported claims.

**Table 2:** Inter- and intra-individual variation in menstrual cycle phase characteristics; the difference between speculation and reality

Speculation	Reality
Eumenorrheic women have consistent menstrual cycle characteristics	Among eumenorrheic women, notable variation in menstrual cycle length and hormone concentrations exist between and within individuals
Most menstrual cycles are 28 days, with ovulation occurring on day 14	It is estimated that this ‘textbook cycle’ represents as few as 10% of all ‘real world’ cycles [30]; cycle length has a healthy range of up to 14 days, such that women can have both short (21 days) and long cycles (35 days). The variability in cycle length is often attributed to changes in the length of the follicular phase, indicated by differences in the timing of ovulation [31]. Ovulation has been detected between days 9 and 21 in confirmed eumenorrheic athletes [29], and even in cycles of the same length (i.e. 28 days), the day of ovulation can have a 10-day range [30]. The timing of ovulation can only be determined through biological testing
Ovulation occurs in all regular cycles (i.e. cycle lengths between 21 and 35 days)	The prevalence of chronic or spontaneous anovulation is up to 20% of all natural cycles [18, 32], which is undetectable without biological testing
Apps can accurately predict menstrual cycle phases without measuring biochemical markers	Even apps with advanced algorithms and access to millions of cycles of data have extremely low predictive accuracy in predicting the day of ovulation (approximately 20% [33])
Wearables and artificial intelligence can accurately represent female physiology	Despite some recent innovative breakthroughs, data from wearables and artificial intelligence should be viewed with extreme caution and are not recommended as substitutes for biochemical markers
Cycle length can be precisely predicted based on the length of past cycles	Large app-based datasets have shown that (1) self-reported cycle length is highly unreliable, (2) more than half of users have an average cycle length variability of more than 5 days, and (3) less than 1% of users have the same cycle length across four consecutive cycles [30]
It is possible to predict menstrual cycle phases by calculating the timing of ovulation based on a predicted cycle length	The act of predicting a highly variable event (i.e. ovulation) using an unpredictable event (i.e. cycle length) as the basis from which to denote menstrual cycle phase introduces an unreasonable level of uncertainty for it to be considered an acceptable method in a scientific setting
In individuals with consistent menstrual cycle length and ovulatory profiles, the concentration of oestrogen and progesterone in each phase is consistent	Even in individuals with consistent menstrual cycle length and ovulatory profiles, the changes in oestrogen and progesterone can vary between individuals and between cycles [34]. That is to say, the hormonal profile of an individual cycle can vary from the ‘textbook’ curve, the mean of the group, or even the profile of the cycle before it. This variation has been elegantly illustrated with individual and group plots by Dam et al. [27] and D’Souza et al. [35] and further challenges the complexity of attributing the observations from an assumed phase to a particular hormonal environment if it has not explicitly been measured
Eumenorrheic women have consistent menstrual cycle characteristics	Among eumenorrheic women, notable variation in menstrual cycle length, day of ovulation, and hormone concentrations exist between and within individuals

## Methodological Quality

The study often used to underpin the rationale for an assumed approach is noteworthy. In 1980, McIntosh et al. [36] examined 88 healthy and mature women aged 23–42 years undergoing fertility treatment, with menstrual cycle lengths of 21–40 days, to develop a predictive dataset on the timing of the luteinising hormone surge. The design and statistical errors employed render their study results questionable at best. Despite methodological guidance to move beyond an assumed predictive approach, some current research continues to employ similar methods to those of McIntosh et al. [36].

Methodological guidelines [16, 25] aimed at increasing the scientific quality of research with female participants have been overlooked, misused, or dismissed in recent publications. These guidelines highlight the importance of verifying cycle phases using biochemical methods, such as urinary ovulation detection kits (which detect the rise in luteinising hormone that occurs prior to ovulation) alongside the direct measurements of ovarian hormones, and importantly the problems of not doing so. Moreover, the British Association of Sport and Exercise Science reiterated these recommendations in an expert statement on conducting and implementing female athlete-based research [37]. Their clear and undisputed reasoning is that, with the exception of menstruation, no other menstrual cycle phases can be reliably identified using an assumed approach. In other words, we would argue that the inter- and intra-individual variability associated with the timing of specific menstrual events (e.g. ovulation), in addition to the prevalence of undetectable subtle menstrual disturbances, render it impossible to know if or when an event took place during a given cycle without biological confirmation. If we are to attempt to relate cycle phases to given outcomes in research, the occurrence and timing of these events/phases must be verified during each cycle of interest. It is time to stop assuming and estimating menstrual cycle phases in research.

## Research Design and Statistical Analysis

The use of assumed or estimated phases involves creating arbitrary boundaries that do not reflect actual biologically meaningful distinctions, which transforms a continuous (i.e. hormone concentration) into a categorical variable. The result is data loss, reduced data granularity, and introduction of assumed independence (i.e. one cannot discern inter- or intra-individual phase-to-phase variability in hormone concentration, resulting in misclassifications when phase boundaries overlap). Overall, this approach results in an introduction of bias where phase definitions may not actually reflect underlying hormonal changes. It is not our intent, nor is it possible, to outline a perfect statistical or research design approach. All researchers should be explicit in their publications about how they have managed such methodological concerns and be transparent about the limitations of the approaches they have undertaken.

## Causality

In research, we are implicitly or explicitly interested in causality. While randomised trials are considered the gold standard to establish causation, experimental manipulation of menstrual cycles is often infeasible and unethical. As a result, studies in this area are typically observational and exploratory. In the absence of additional information and evidence, these exploratory findings can be valuable for developing some individual recommendations, while also acknowledging their tentative nature. However, due to their inherent limitations, such data should primarily be used for hypothesis generation and identifying areas for further investigation, rather than as universal blueprints for all female athletes. Using assumed or estimated menstrual cycle phases undermines the validity of this research, as observations are based on an unconfirmed status, making it challenging to draw reliable causal inferences.

## Field-Based Research

The methodological considerations for identifying menstrual cycle phases in research are covered elsewhere [16, 25]. Research within elite sport (i.e. field-based research) is challenging. Athlete priorities and availability, coach/practitioner engagement, and scarcity of resources often mean that research is not a priority. The argument for using assumed or estimated phases, rather than direct verification in field-based research, is often based on pragmatism and practicalities, stating that assumed or estimated phases are non-invasive, time efficient, and easier for athlete-participants to engage with. Although these points are not disputed, they pose an important question. Should we accept a method that is simpler to execute if it is unable to reliably answer the proposed research question? Our answer is a resounding no since the trade-off is too high. This approach is at the expense of research quality/scientific rigour and generates outputs that are less meaningful and less likely to move female athlete science and medicine forward. We maintain that verified phases are feasible and required in field-based research.

The reality is that field-based research requires a balance between feasibility and acceptability. We need to be able to accept certain limitations in research design to make it possible in the practical setting. However, an unacceptable scenario occurs when there is strong scientific evidence that the proposed methods are not valid or reliable. We observe that some studies, due to the challenges of field-based research, adopt a convenient optimism in interpreting their data, which downplays the mis-categorisation of observed effects, and is a misguided practice. Moreover, we contend that field-based research related to menstrual cycle phases, using athletes as participants, should be undertaken or supervised by suitably qualified individuals with the relevant knowledge and experience of ovarian hormone profiles and high-quality research standards. It should be noted that the guidance contained herein is not intended to be considered as a panacea for all research studies using female athletes as participants; rather it is intended to aid, in part, those looking to expand their knowledge and understanding of establishing menstrual cycle phases in research settings.

Athlete, staff, and financial burdens, alongside challenges of biological sample collection and storage and coaching staff buy-in, have been used as justification for an assumed or estimated phase approach in field-based studies. In response we:

- Challenge ‘athlete and staff burden’ on the basis that these direct measurements are no more or less intensive than other routine measurements taken in applied research. The importance of these direct measurements to athletes and staff must be considered, using an opt-in, rather than a mandated, approach for those who are keen to take part in such protocols. We must interrogate the accuracy and usability trade-off of all cycle phase direct measurements, noting that assumed or estimated phases are not direct measurements and would not feature in this matrix.
- Question the actual—rather than perceived—financial burden of taking, storing, and analysing biochemical samples. We acknowledge the cost associated with techniques such as urinary ovulation detection kits and blood samples for determination of progesterone; however, we assert that these procedures are not needed on an ongoing continual basis. Instead, they can be used at key strategic times during a study. In addition, we recognise that not all research studies can afford even periodic biochemical assessments, which is why suitable alternatives need to be developed without losing the scientific integrity of data production, interpretation, and reporting.

In addition, we must remember that athletes are not automatic research participants. We need to consider the ethical implications of exposing athletes to methods (i.e. data collection) or interventions (i.e. application of findings) that may be scientifically unsound or proposed because of spurious conclusions. Athletes must be made aware of the limitations, rationales, and potential for unintended findings (e.g. the discovery of menstrual dysfunction, pregnancy, etc.) of all approaches used within the research consent process.

## Interpretation and Implementation of Data

The purpose of many studies in sport-related research is translation. On this basis, we are resolute that it is unacceptable to guess/estimate ovarian hormone concentrations and then offer suggestions based on those guesses. Undoubtedly, women’s sport is a current and welcomed ‘hot topic’ in sport-related research; however, are we willing to sacrifice quality and credibility for convenience (i.e. research volume) and incorrect conclusions? We have a duty of care to athletes and a responsibility to science and medicine to ensure that athlete care initiatives are developed from research of the highest possible quality; assumed menstrual phases are not a zero-risk approach. Using assumed phases to declare a participant eumenorrheic based solely on the presence of menses risks unintentionally failing to identify anovulation or luteal phase deficiency, thus misrepresenting and skewing the characteristics of the sample. For instance, injury surveillance studies that have use assumed phases to associate injury incidence to certain cycle phases risk distracting athletes, coaches, and practitioners from better-evidenced insights and subsequent prevention strategies, as well as the misdirection of scarce time, resources, and effort. When examining menstrual cycle phases from a research perspective, accurate assessment, interpretation, and reporting of these phases is non-negotiable.

Alongside our appeal to researchers to reject assumed or estimated menstrual phases, we urge athletes, practitioners, funding bodies, sport organisations, the media, and other stakeholders to employ due diligence when identifying research examining the effects of menstrual cycle phases on any given outcome. We also ask journals and other publications to maintain rigorous standards when reviewing and accepting papers on women athletes, avoiding a laxer approach to meet quotas for female-based research outputs. We accept that determining the quality of research can be difficult, a rationale for this piece, which we hope will dispel some of the current myths associated with the determination of menstrual cycle phases in laboratory and field-based research. Studies must not be used to misdirect athletes, practitioners, and other end-users on the reality of cycle phase-based data collected in either laboratory or field-based settings.

Undoubtedly, studies employing assumed or estimated menstrual cycle phases result in more publications involving women, who have historically been under-represented in sport-related research [38, 39]. Nonetheless, as researchers, we must consider not only the benefits of growing research but also the risk of generating weak or spurious evidence, which can be more harmful than having limited research. We should consider the repercussions of low-quality evidence replacing little evidence, resulting in misdirected practices that are independent of the strength of evidence. In elite sport, data analysis is critical for high performance. We should make sure that sound data, without assumptions, are used for the determination of menstrual cycle phases.

## Conclusion

Assumed or estimated menstrual cycle phases are an unreasonable approach in research studies. When only menstruation is tracked, the menstrual cycle can only be divided into menstruation and non-menstruation days. At a minimum, ovulation needs to be confirmed and biochemical luteal phase deficiency excluded to establish menstrual cycle phases, which are based on significant changes in ovarian hormone concentrations. Given the issues associated with assumed or estimated phases described herein, we suggest that researchers, amongst others (e.g. reviewers, editors, the media), consider the individual and cumulative value of these studies on women’s sport, and either reject these methods, or at the very least use transparent and honest reporting about inadequacies of approaches taken. We encourage researchers to reflect upon the issues described herein and to consider the best direction for future research (Table 1).

## Data Availability

Since this is a Current Opinion article and not original research, there are no data associated with it.

## Declarations

### Equity, Diversity and Inclusion Statement

Our author team consists of ten female and eight male authors, representing early-career, mid-career, and senior-researchers, clinicians, and practitioners from elite international, professional, and Olympic sports, from seven countries and three continents. The senior corresponding author is a woman.
